# Genome sequencing and analysis of plant growth-promoting attributes from **Leclercia adecarboxylata**

**DOI:** 10.1590/1678-4685-GMB-2020-0130

**Published:** 2021-01-27

**Authors:** Aline Snak, Eliane Cristina Gruszka Vendruscolo, Marise Fonseca dos Santos, Adriana Fiorini, Dany Mesa

**Affiliations:** 1 Universidade Federal do Paraná, Labiogen-Laboratório de Bioquímica e Genética, Palotina, PR, Brazil.; 2 Universidade Federal do Paraná, Departamento de Biociências, Palotina, PR, Brazil.; 3 Universidade Federal do Paraná, Departamento de Bioquímica, Centro Politécnico, Jardim das Américas, Curitiba, PR, Brazil.

**Keywords:** Endophyte, Leclercia, genome, plant growth promotion, strains

## Abstract

Plant growth-promoting bacteria are ecological alternatives for fertilization, mainly for gramineous. Since plant x bacteria interaction is genotype and strain dependent, searching for new strains may contribute to the development of new biofertilizers. We aim to characterize plant growth-promoting capacity of *Leclercia adecarboxylata* strain Palotina, formerly isolated by our group in corn. A single isolated colony was taken and its genome was sequenced using Illumina technology. The whole genome was compared to other *Leclercia adecarboxylata* strains, and their biological and growth-promoting traits, such as P solubilization and auxin production, were tested. Following that, a 4.8 Mb genome of *L. adecarboxylata* strain Palotina was assembled and the functional annotation was carried out. This paper is the first to report the genes associated with plant growth promotion demonstrating *in vitro* indole acid production by this strain. These results project the endophyte as a potential biofertilizer for further commercial exploitation.

## Introduction

*Leclercia adecarboxylata*, a member of the Enterobacteriaceae family*,* is a motile, aerobic, omnipresent Gram-negative bacterium. Infections by *L. adecarboxylata* in humans are scarcely reported, being considered an opportunistic pathogen (Kashani *et al.*, 2014; Hoyos-Mallecot *et al.*, 2017; Choudhary *et al.*, 2018). This strain was first described and named *Escherichia adecarboxylata* by Leclerc (1962), and, later received the generic name Leclercia from Tamura *et al.*, (1986). It was phenotypically differentiated by biochemical and DNA hybridization assays from other Enterobacteriaceae species (Choudhary *et al.*, 2018). Recently, Hoyos-Mallecot *et al.* (2017) published the draft genome of *L. adecarboxylata* strain harboring an NDM1 (Multidrug-resistant New Delhi metallo-β-lactamase 1) gene.

Plants and microorganisms naturally interact in the soil, forming a narrow and complex communication network. This network operates on biochemical to molecular signals, which can be altered according to the type of association (Souza *et al.*, 2015). The promotion of direct growth occurs through the availability of nutrients, nitrogen, phosphate, as well as the production of plant regulators as auxins, cytokinins and amino acids. These regulators mainly promote central and lateral root growth, increasing the absorption surface, which in turn increases the root’s nutrient and water uptake (Beneduzi *et al.*, 2012; Jha and Saraf, 2015).

However, the promotion of indirect growth occurs by means of induced systemic resistance (ISR). Some biocontrol mechanisms of pathogens are antibiosis, parasitism, competition for nutrients, production of hydrogen cyanide, siderophores, including the ones involved in responses to abiotic stresses, such as drought, salinity, extreme temperatures (Moreira *et al.*, 2016).

Although this organism has been reported globally in food, water and animals (Tamura *et al.*, 1986; Anuradha, 2014), for instance in strawberry root (Laili *et al.*, 2017), evidences of its efficiency as plant growth promoter bacteria is scarce. In this context, we sequenced the complete genome of *Leclercia adecarboxylata* strain Palotina carrying out a comparative analysis with genomes of 16 different strains. This study provides new insights into genetic determinants, and as such may clarify some reported metabolic abilities of the Palotina strain, offering basic information on genetic plant growth promotion that may be relevant for biotechnological interest.

## Material and Methods

### DNA extraction and sequencing

Genomic DNA was extracted from the isolated strain following the protocol by Souza *et al.* (1991), using as template for a PCR reaction, Y1 and Y3 primers for amplification of the 16S rRNA gene (Cruz *et al.*, 2001). Amplicons were enzymatically treated with ExoI/SA and the sequencing was performed on BigDye® Terminator v3.1 Cycle Sequencing in an ABI3500xL. The resulting sequences were assembled with CAP3 using BLASTn for comparison at NCBI.

The gDNA of *Leclercia* was quantified with Qubit, diluted and used for the construction of genomic DNA sequencing libraries using Illumina NexteraXT kit, according to the manufacturer's recommendations. The libraries were quantified and the quality was verified by means of Bioanalyzer. The libraries were diluted to 500 pM and pooled. This pool was quantified by qPCR using the Kapa Biosystems kit, and 17.5 pM of pooled libraries were sequenced in the Illumina MiSeq with 500V2 kit in paired-end, generating paired reads of 250 base pairs from DNA fragments.

### Genome assembly, annotation and serotyping

Overall, 5,795,728 reads were generated, representing a 31-fold coverage for the strain Palotina. FastQC (www.bioinformatics.babraham.ac.uk/projects/fastqc/) was used to check the quality of the reads. SPAdes program (Bankevich *et al.*, 2012), version 3.11.1 was used to reassemble the sequence dataset, which were deposited at NCBI site under the BioSample access number SAMN09791487. In order to identify putative coding sequences (CDS) and provide an initial automatic annotation, the genome sequences were submitted to the RAST server annotation pipeline (Aziz *et al.*, 2008) and Artemis (Sanger Institute, Cambridge, UK) was used to curate annotations manually.

### Comparative genomics

BLAST Ring Image Generator (BRIG) program (Alikhan *et al.*, 2011) was used to compare the genome of *L. adecarboxylata* strain Palotina at nucleotide level against other strains available in the NCBI site (Table 1). It uses the Basic Local Alignment Search Tool (BLAST) (Altschul *et al.*, 1990), which is considered the most common tool for comparing genomes. Only 16 complete genomes found at NCBI were considered, being 9 from *L adecarboxylata* strains (NCTC13032; Z96-1; E61; P12375; J656; 16005813; USDA-ARS-USMARC-60222; E1 and R25) and 7 from *Leclercia* sp. strains (W6; 119287; 1106151; LSNH1; LSNH3; J807 and W17), the great majority from clinical isolates (Table 1).

**Table 1.** Characteristics of *Leclercia* strains used in the genomic comparison.

Organism	Strain	BioSample	Assembly	Size (Mb)	GC%	Replicons	Isolation source
*Leclercia adecarboxylata*	NCTC13032	SAMEA2580321	GCA_901472455.1	5.06	55.5	Chromosome	Drinking water
*Leclercia adecarboxylata*	Z96-1	SAMN11950933	GCA_006171285.1	5.76	55.4	Chromosome + 7 plasmids	Human stool
*Leclercia adecarboxylata*	E61	SAMN12289350	GCA_008931385.1	5.69	55.0	Chromosome + 5 plasmids	Shower
*Leclercia adecarboxylata*	P12375	SAMN13341565	GCA_009720165.1	4.93	55.6	Chromosome	Hospital
*Leclercia adecarboxylata*	J656	SAMN12530229	GCA_008807335.1	4.84	55.6	Chromosome	Human secretion
*Leclercia adecarboxylata*	16005813	SAMN10923138	GCA_004295325.1	4.82	55.7	Chromosome	Sputum
*Leclercia adecarboxylata*	USDA-ARS-USMARC-60222	SAMN04158503	GCA_001518835.1	4.80	55.8	Chromosome	Calf nasopharynx
*Leclercia adecarboxylata*	E1	SAMN12289304	GCA_008931445.1	5.51	54.8	Chromosome + 6 plasmids	Shower
*Leclercia adecarboxylata*	R25	SAMN10790527	GCA_006874705.1	4.91	56.2	Chromosome + 2 plasmids	Rabbit
*Leclercia sp.*	W6	SAMN09667310	GCA_003336345.1	4.95	55.9	Chromosome	Human stomach
*Leclercia sp.*	119287	SAMN13394079	GCA_009734485.1	4.87	55.6	Chromosome	Hospital
*Leclercia sp.*	1106151	SAMN13394552	GCA_009740165.1	4.85	56.1	Chromosome	Urine
*Leclercia sp.*	LSNIH1	SAMN06040403	GCA_002902985.1	5.41	55.5	Chromosome + 4 plasmids	Sludge
*Leclercia sp.*	LSNIH3	SAMN06040408	GCA_002935105.1	5.39	55.3	Chromosome + 4 plasmids	Sludge
*Leclercia sp.*	J807	SAMN13393390	GCA_009734465.1	4.72	56.1	Chromosome	Human blood
*Leclercia sp.*	W17	SAMN09667311	GCA_003336325.1	5.13	56.0	Chromosome + 2 plasmids	Human stomach

Also, three genes related to plant growth promotion (P metabolism and auxins biosynthesis) were selected and compared by BLASTn against all genomes. In phylogenetic analyses, a Neighbor-Joining tree (Saitou and Nei, 1987) was constructed with 98 genomes with NCBI Tree Viewer (version 1.17.5).

### **Biochemical characterization of *L. adecarboxylata***

At first, *L. adecarboxylata s*train Palotina was isolated in LB medium, growing well in DYGS (Dobereiner *et al.*, 1995), following the isolation protocol by (Chaves *et al.*, 2019). Visual assays determined bacteria morphology and colony color. Bacterial phosphate solubilization was detected *in vitro* by inoculation in NBRIP medium (Nautiyal *et al.*, 2000). A bacterial colony was collected with a toothpick, and each ¼ plate of NBRIP medium plate was inoculated. A halo around the colonies was observed after 10 days in a culture incubated at 28 °C. The solubilization index (SI) was calculated as: SI=Diameter of Halo (mm) / Diameter of colony (mm) (Nautiyal, 1999).

Indole-3-acetic acid (IAA) production by bacteria was based on the Glickmann and Dessaux (1995) protocol. Isolates were inoculated into glass vials (penicillin-type) containing 4 mL of medium with tryptophan (5.0 g.L^-1^ glucose, 0.025 g.L^-1^ yeast extract and 0.204 g.L^-1^ L-TRP) and no tryptophan supplementation (Sarwar and Kremer, 1995). Triplicate vials were incubated in a shaker cooled at 28 °C in the dark at 120 rpm. After the growth, which occurred in 48 h, 2 mL of the culture medium was centrifuged at 10000 g for 10 min at 4 °C. Next, 1 mL of the bacterial suspension supernatant was transferred to a 15 mL Falcon-type tube with the addition of 1 mL of Salkowski reagent. The standard curve was assayed for final concentrations of 0 to 0.03 mg mL^-1^. Samples were left in the dark for 30 min and the AIA quantification was performed by spectrophotometer reading at 535 nm.

*L. adecarboxylata* was primarily screened and further grown on LB plate containing 1%, 2%, 5% and 10% NaCl separately for 48 h at 30 °C. In addition, the optimum pH was checked using LB liquid medium with different pH (4; 5; 5.5; 6; 6.5; 7; 7.5 and 8). The growth temperatures assessed were 25 and 37 ^o^C. The presence of oxidase was tested using TEMED 1 % (N-N-dimetil-p-phenilenediamine) (Kovacks,1956). The presence of catalase was verified by the presence of bubbles when hydrogen peroxide was deposited in a colony (Yano *et al.*, 1991). All assays were made in triplicate.

Blood agar plates (5 % (v/v) sheep blood) were used for biosafety test (Russell *et al.*, 2006; Suleman *et al.*, 2018). The hemolytic capacity was evaluated after 48 h from fresh culture of *L. adecarboxylata* streaked onto blood agar plates and incubated at 37 ± 2 ^o^C.

## Results

### Genome assembly, annotation and comparative genomics

**Figure 1 -:**
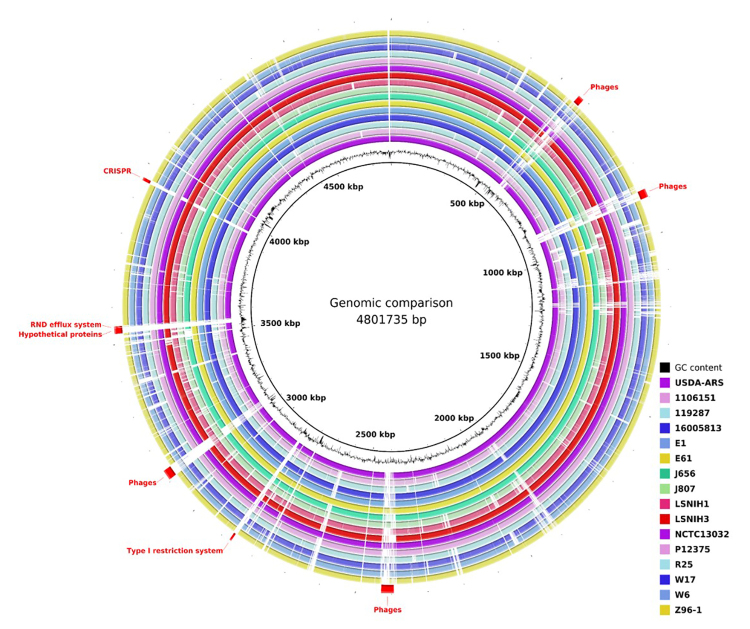
Genomic comparison among *Leclercia* strains. Each ring represents the genome of one strain. *Leclercia adecarboxylata strain Palotina* (NCBI BioSample SAMN09791487) was used as genome of reference. Gaps in the rings mean absence of the region in the target genome.

After *de novo* assembly, the genome of *Leclercia adecarboxylata* strain Palotina was represented in 20 contigs, sized 4,801,735 bp, with GC content of 55.7%, 4.379 coding sequences and no plasmid were observed. The comparison showed differences in the genome of *L. adecarboxylata* strain Palotina (Figure 1, Table 1). The size of *L. adecarboxylata* strains ranged from 4.72 to 5.76 Mb, their CG content between 55-56% CG content. Some contained plasmids (up to 7). The BRIG genomic analyses showed CRISPR system and mobile elements as phages were absent in some strains. One interesting data is that no *Leclercia* sp contained the indole acetamide hydrolase gene. However, group genes related to bacterial systems, such as several hypothetical proteins, Type I restriction and cobalt/cadmium/zinc RND efflux, were absent in all strains used in the comparison (Figure 1, Figures S1 to S3).

**Figure 2 -:**
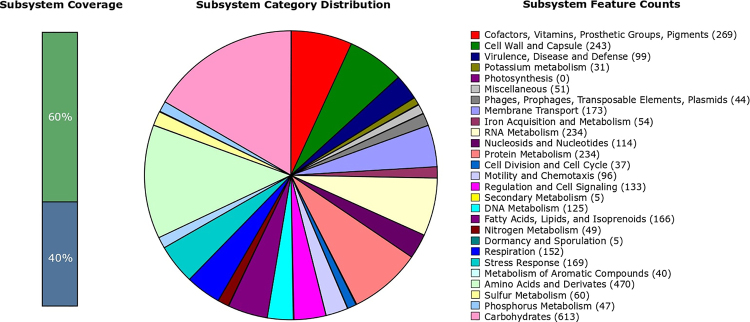
Functional analyses of *L adecarboxylata strain Palotina* genome.

Of all identified coding sequences, Rast server classified 2597 genes (60%) in categories (Subsystems) and 1782 (40%) were grouped as not classified (Not in Subsystems). The categories with the highest number of genes were carbohydrates metabolism (613 genes), followed by amino acids and derivatives (470 genes), and protein metabolism with 302 genes (Figure 2). Dormancy, sporulation and secondary metabolism showed the lowest gene number (only 5).

In addition, in the nitrogen metabolism category 49 genes were identified and then grouped in four subcategories: nitrosative stress (6 genes), nitrate and nitrite ammonification (22 genes), Ammonia assimilation (13 genes) and denitrifying reductase gene clusters with 8 genes. Phosphorus metabolism (47 genes) had 8 genes related to PHO regulon and high affinity phosphate transporter, Phosphate metabolism (22), Polyphosphate (3) and Alkylphosphonate utilization (14). Finally, in secondary metabolism 5 genes were related to Auxin biosynthesis (Table S1).

**Figure 3 -:**
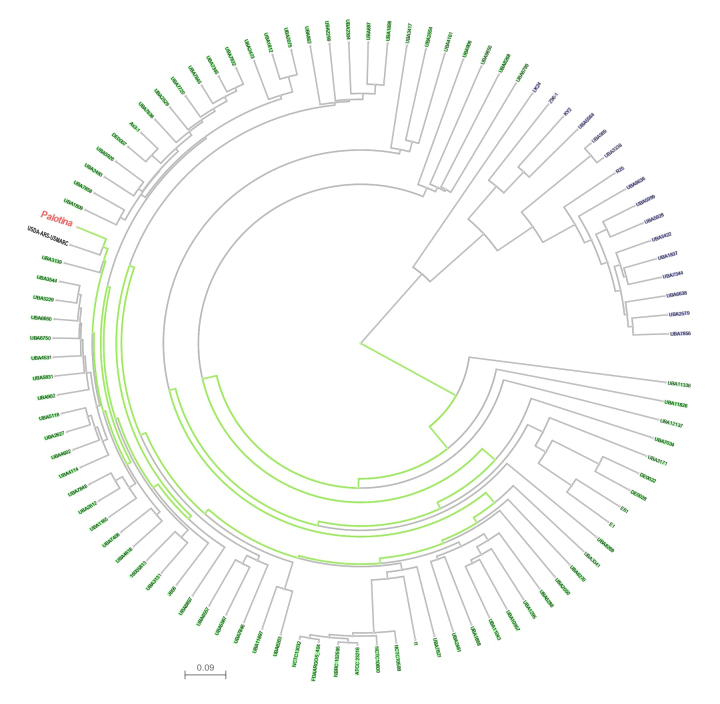
Phylogenetic relationship among *Leclercia* strains. In red **L. adecarboxylata** strain Palotina. The sequences were aligned using the Neighbor-joining method (Saitou and Nei, 1987). The 1,000 resampling bootstrap values are shown.

BLASTp comparison revealed 14 genes related to plant growth promotion that showed high identity (> 97%) and high e-value (Table S2). The phylogenetic analysis of all the 98 genomes found at NCBI, belonging to “*Leclercia*”, showed a higher similarity between *L. adecarboxylata* strain Palotina and USDA-ARS-USMARC-60222, isolated from calf nasopharynx, an indicative that these bacteria can be associated to agricultural area, unlike clinical strains (Figure 3).

### **Biochemical and molecular characterization of *L. adecarboxylata***

The biological and plant growth promotion traits are summarized in Table 2. *L. adecarboxylata* strain Palotina is a cream rod-shaped, non-spore-forming, motile, Gram-negative bacillus of family Enterobacteriaceae, having an optimum pH growing range between 5.0-8.0, 25-37 ^o^C for growth temperature and a low salinity toleration (below 5 %).

**Table 2.** Biological and plant growth promotional properties of *L. adecarboxylata* strain Palotina.

Attributes	
pH tolerance levels	5.0-8.0
Optimum pH for growth	6.0-7.0
NaCl tolerance	< 5%
Optimum temperature for growth	25-37^o^C
IAA production	Positive (2.6 ± 0.3 µg.mL^-1^)
Phosphate solubilization	Positive (PSI>2)
Oxidase	Negative
Catalase	Negative
Chitinase	Negative
Hemolysis	Positive

The strain also presented oxidase and negative catalase response (Table 2). In addition, genes for chitinase production were found in the genome. Antifungal resistance was not tested in *L. adecarboxylata* strain Palotina by the inoculation with *Aspergillus flavus*.

Halo zone formation on blood agar medium was observed *in vitro*, which points to hemolysin gene expression*,* confirming the opportunistic pathogen trait. A lipase gene was annotated in the genome, demonstrates a potential use of this strain for biotechnological purposes. Moreover, we identified genes that can be related to the improvement of nutrient availability to plants (Tables 3 and 4), which is consistent with many plant growth promoting bacteria (PGPB). The genome of *L adecarboxylata* strain Palotina possesses genes encoding glucose dehydrogenase (*gcd*), the major enzyme responsible for the production of gluconic acid. Palotina strain showed a medium capacity of P solubilization (2 < PSI > 4) (Table 2). UDP-glucose dehydrogenase gene was present in all 16 *Leclercia* genomes (Table 3).

**Table 3.** Plant growth promotion genes in all strains compared.

		Identities %			
Organism	Strain	Indoleacetamide hydrolase	UDP-glucose dehydrogenase	Phosphoribosylanthranilate isomerase	Isolation source
*L. adecarboxylata*	NCTC13032	98	98	99	Drinking water
*L. adecarboxylata*	Z96-1	79	88	90	Human stool
*L. adecarboxylata*	E61	98	98	98	Shower
*L. adecarboxylata*	P12375	98	98	98	Hospital
*L. adecarboxylata*	J656	98	97	99	Human secretion
*L. adecarboxylata*	16005813	97	98	98	Sputum
*L. adecarboxylata*	USDA-ARS-USMARC-60222	98	98	99	Calf nasopharynx
*L. adecarboxylata*	E1	98	98	98	Shower
*L. adecarboxylata*	R25	87	92	91	Rabbit
*Leclercia sp.*	W6	Not match	88	90	Human stomach
*Leclercia sp.*	119287	87	92	91	Hospital
*Leclercia sp.*	1106151	Not match	89	90	Urine
*Leclercia sp.*	LSNIH1	Not match	89	90	Sludge
*Leclercia sp.*	LSNIH3	98	99	99	Sludge
*Leclercia sp.*	J807	Not match	89	90	Human blood
*Leclercia sp.*	W17	Not match	89	90	Human stomach

*Trp* cluster (*trpC, D* and *F*), tryptophan-permease, tryptophan-synthase (a) and (b) genes, and indole acetamide hydrolase gene involved in tryptophan biosynthesis were found in the genome (Table 4). We observed an increase of 2.3-fold in IAA production when tryptophan was added to the culture medium (Table 2). When we compared all 16 *Leclercia* genomes, the phosphoribosyl anthranilate isomerase gene was found in all strains (Table 3).

**Table 4.** List of genes attributable to plant growth promotion traits in *L adecarboxylata* genome.

Plant growth promotion traits	Genes with potential for conferring PGP traits
Phosphate solubilization	Glucose dehydrogenase gene
IAA production	*TrpD, TrpF*, tryptophan-permease, tryptophan-synthase (a) and (b), indole acetamide (Indole acetamide hydrolase)
N assimilation	*GS* type I, NaDPH-GOGAT, *Amt*, NRI, *PIIK*
Siderophore production	Ferric hydroxamate ABC transporter (*Fhu* genes)*, ViuB, TonB, TonB3, FiU*
Acetoin & butanediol synthesis	Acetolactate synthase large subunit, Acetolactate synthase small subunit
Phenazine production	*phzF*
Chitinase production	Chitinase gene
Trehalose metabolism	Trehalose -6-phosphate synthase gene
Quorum sensing	Autoinducer 2 (AI-2) transport and processing (*lsrACDBFGE* operon) N-3-oxohexanoyl-L-homoserine lactone quorum-sensing transcriptional activator
Heat shock proteins	*groE, YciM, hslJ, FtsJ/RrmJ*
Cold shock proteins	*cspA, C, D, E, G*
Glycine-betaine production	*proX*
Peroxidases	*osmC*, glutathione peroxidase genes similar to *Enterobacter asburiae*
Catalases	Catalase gene
Superoxide dismutase	superoxide dismutase gene
Auxins production	Monoamine oxidase, Phosphoribosyl anthranilate isomerase, Tryptophan synthase alpha and beta chain

Ammonia assimilation genes, among others, seem to be the main N metabolism pathway, confirmed by the presence of several genes, such as *GS type I* (Glutamine synthase); *NADPH-GOGAT*; *Amt* (ammonia transporter); *NRI* (protein regulator) and *PKII* (Table 4). 

The Ferric hydroxamate ABC transporter *Fhu* genes *Fhu, ViuB, TonB, TonB3, FiU* were verified, although we did not evaluate the siderophore production (Table 4). Genes coding for antioxidant enzymes as peroxidases, catalases, superoxide dismutase, among others, were found at *L. adecarboxylata* genome (Table 4). Genes that enable bacteria to survive at harsh conditions were also detected: heat shock tolerance genes (*groE, YciM, hslJ, FtsJ/RrmJ*), cold shock tolerance (*cspA, C, D, E, G*), and glycine betaine (Gupta *et al.*, 2014).

Some genes related to cell-cell communication via quorum sensing (QS) were found in the *L. adecarboxylata* genome: N-3-oxohexanoyl-L-homoserine lactone quorum-sensing transcriptional activator, Autoinducer 2 (AI-2) transport and processing (*lsrACDBFGE*) operon (Table 4).

## Discussion

Our findings indicate the complete absence of the RND protein family, which was reported as a group of bacterial transport proteins involved in cell division, nodulation and heavy metal resistance (Nies, 2003). Another gene sequence that appeared to be distinct among *Leclercia* genomes is the clustered regularly interspaced short palindromic repeats (CRISPR), which is related to the microbial immune system. It contains a family of proteins whose functional domains are related to polynucleotide-binding proteins, polymerases, nucleases, and helicases (Horvath and Barrangou, 2010; Ishino *et al.*, 2018). This region was observed in only three of *Leclercia* strains, including strain Palotina, which shows a horizontal gene transfer promoting a genomic differentiation among strains (Portillo and Gonzalez, 2009). A Type I restriction system or Restriction modification system (R-M system) was absent in all compared genomes. R-M system has large pentameric proteins with separate restriction, methylation and DNA sequence-recognition subunits (Loenen *et al.*, 2014), which grants to the host bacterium a selective advantage (Sitaraman, 2016).

Carbohydrate metabolism genes were present in *L. adecarboxylata* strain Palotina enabling this bacterium to grow in different media using different carbohydrate/energy sources, including root exudates and other organic compounds. Moreover, this strain would interact positively with plants under harsh soil conditions.

Although our strain was able to carry out an alpha hemolysis, Muratoglu *et al.,* (2009) and Anuradha (2014), who tested *L. adecarboxylata* Ld1 and human isolates respectively, found a negative response to blood hemolysis. The contrasting results could possibly be explained by the presence of the hemolysin gene set found in the genome of our strain.

Strain Palotina showed a P solubilization capacity, probably explained by the presence of the *gcd* gene. Glucose dehydrogenase is the key enzyme in the biosynthesis of gluconic acid in the direct oxidation pathway of glucose, responsible for P solubilization (Chen *et al.*, 2016; Suleman *et al.*, 2018). The amount of gluconic acid released would control the availability of soluble phosphates (De Werra *et al.*, 2009). Also, UDP‐glucose dehydrogenase found in all compared strains (Table 3) catalyzes an NAD^+^‐dependent two-fold oxidation of UDP‐glucose to generate UDP‐glucuronic acid (Chen *et al.*, 2019). This acid is also a precursor to UDP‐xylose component of the cell wall polysaccharides in plants (Gibeaut and Carpita, 1994).

Another important PGP feature is the auxins/cytokinins biosynthesis. The biosynthesis of IAA occurs from tryptophan (Patten *et al.*, 2013) and a higher IAA production can be induced by addition of tryptophan to culture media. Biosynthesis of tryptophan encoded by *trp* genes occurs in five-step reactions from chorismate (Spaepen and Vanderleyden, 2011). Five different pathways were described to the IAA production in bacteria: the indole-3-acetamide (IAM), indole-3-pyruvic acid (IPyA), indole-3-acetonitrile (IAN), tryptamine (TAM), and tryptophan side-chain oxidase (TSO) pathways (Kochar *et al.*, 2013; Li *et al.*, 2018).

Genomic analyses identified the indole acetamide hydrolase gene, which explains the IAA production mainly by IAM pathway suggesting the tryptophan-dependent IAM pathway function in strain Palotina. The main pathway to IAA production in PGPB is via indole-3-pyruvic acid, dependent on L-tryptophan (Souza *et al.*, 2015). Not all *Leclercia adecarboxylata* and no *Leclercia* sp. strains present the indole acetamide hydrolase gene, which suggests that this gene has been acquired. This fact explains the association between bacteria and corn plants.

In addition, we identified the sequence of phosphoribosyl anthranilate isomerase (PRAI) encoded by *trpC* (Table 4). This enzyme is responsible for the conversion of N-(5′-phosphoribosyl)-anthranilate (PRA) to 1-(o-carboxyphenylamino)-1-deoxyribulose 5-phosphate (CdRP), the fourth step in tryptophan biosynthesis (Thoma *et al.*, 2000). Moreover, monoamine oxidase plays an important role in tryptamine biosynthesis, whose oxidative deamination of tryptamine to indole acetaldehyde is known to be the main course for IAA formation, despite the fact that the role of monoamine oxidase has not been completely characterized (Ueno *et al.*, 2003). The presence of these genes suggests that the tryptophan-dependent IAM and TAM pathways function in *L. adecarboxylata*.

*L adecarboxylata* produced 2.6 µg.mL^-1^ of IAA (Table 2). Albeit the variable levels, Gupta *et al.,* (2014) related IAA production of 1.2-2.5 ug.mL^-1^ to candidate PGPB strains isolated from coconut, cocoa and arecanut plants, while Moreira *et al.,* (2016) found strains that could produce more than 80 µg.mL^-1^ of indolic compounds. We did not identify an *acdS* gene coding for ACC deaminase enzyme in our strain, which demonstrates the absence of this enzyme among PGP traits. However, Kang *et al.* (2019) suggested that the IAA and ACC deaminase helped tomato (*Solanum lycopersicum*) plants to tolerate salt stress, despite having found *acdS* gene in *L. adecarboxylata* strain MO1.

Ammonia assimilation, among others, seems to be the main N metabolization pathway from nitrate. In addition, this strain exhibits the genes for denitrification used as energy source. These genes indicated that *L. adecarboxylata* has an important role in soil N cycling system. The results agree with Muratoglu *et al.* (2009) who observed an absence of nitrogen fixation capacity as well as a presence of NO_2_ metabolism in Ld1 strain. From these data, *L. adecarboxylata* can be used as a model for PGP bacteria exclusively by auxins production.

Peroxidases, catalases, superoxide dismutase, and glutathione transferases genes found at *L. adecarboxylata* genome could help plants to overcome oxidative stress. Also, heat and cold shock genes could support bacteria to survive during abiotic or biotic stress (Gupta *et al.*, 2014), which enable bacteria to adapt to adverse growth conditions. 

Another strategy to copy with abiotic stresses is the accumulation of compatible solutes, such as trehalose, proline and glycine betaine, among others, by some soil bacteria (Suarez *et al.*, 2019). The strain Palotina genome contains trehalose-6-phosphate synthase involved in GDP- or UDP-glucose conversion to trehalose (Avonce *et al.*, 2006). Also, glycine betaine/proline betaine-binding periplasmic protein (*ProX*) is one of three genes from operon *VWX* involved in binding compatible solutes with high affinity and specificity (Schiefner *et al.,* 2004).

We also found genes related to acetoin and 2,3 butanediol production, which are volatile compounds (VOCs) involved in plant growth bacteria/fungi interaction as acetolactate synthase large and small subunit (Yi *et al.*, 2016; Fincheira and Quiroz, 2018). VOCs are synthesized by the condensation of two pyruvate molecules into acetolactate by acetolactate synthase, which forms acetoin by acetolactate decarboxylase decarboxylation. The reduction of acetoin by acetoin reductase results in 2,3-butanediol (Suarez *et al.*, 2019). 

The strain Palotina contains *phzF* encoding phenazine biosynthesis. Phenazines can modify the cellular redox state by electron transport, acting in the cell signaling regulating gene expression. By contributing to biofilm formation and architecture, it can enhance bacterial viability in the rhizosphere (Pierson and Pierson, 2010).

Autoinducer 2 (AI-2) transport and processing (*lsrACDBFGE*) operon (Table 4) codifies molecules related to motility, biofilm formation and production of virulence factors (Reading and Sperandio, 2006). AI-2 has been suggested to act directly through quorum sensing while (*lsrACDBFGE*) operon encodes an ATP-binding cassette transporter (ABC transporter) that internalizes AI-2 in gram-negative bacteria (Papenfort and Bassler, 2014). In the marine bacterium *Vibrio fischeri*, N-(3-oxo-hexanoyl)-L-homoserine lactone (3-oxo-C6-HSL) acts as autoinducer in the quorum-sensing system (Yan *et al.*, 2007).

Genome sequencing of a strain might provide more abundant screening tools for the PGPB, which could be readily detected in genomes (Finkel *et al.*, 2017). The authors mentioned that the presence of minimal *Nif cluster* and genes required for indole acetic acid production are potent markers, albeit at variable levels, for screening potential strains, making the process faster and less labor extensive. Scagliola *et al.* (2016) affirmed that a potential PGPB candidate must have the ability to solubilize phosphate and iron (siderophores) and IAA. The data pointed to a PGP strain candidate and further studies should be conducted to reveal the full genetic mechanisms of plant interaction.

## Supplementary Material

The following online material is available for this article:

Table S1 - List of genes and their function in *L. adecarboxylata* strain Palotina.

Table S2 - List of genes related to plant growth promotion in *L. adecarboxylata* strain Palotina.

Figure S1 - Genomic map of CRISPR system.

Figure S2 - Genomic map of RND efflux system.

Figure S3 - Genomic map of Type I restriction-modification system.

## References

[B1] Alikhan NF, Petty NK, Ben Zakour NL, Beatson SA (2011). BLAST Ring Image Generator (BRIG): Simple prokaryote genome
comparisons. BMC Genomics.

[B2] Altschul SF, Gish W, Miller W, Myers EW, Lipman DJ (1990). Basic local alignment search tool. J Mol Biol.

[B3] Anuradha M (2014). Leclercia adecarboxylata isolation: Case reports and
review. J Clin Diagnostic Res.

[B4] Avonce N, Mendoza-Vargas A, Morett E, Iturriaga G (2006). Insights on the evolution of trehalose
biosynthesis. BMC Evolut Biol.

[B5] Aziz RK, Bartels D, Best AA, DeJongh M, Disz T, Edwards RA, Formsma K, Gerdes S, Glass EM, Meyer F (2008). The RAST Server: Rapid Annotations using Subsystems
Technology. BMC Genomics.

[B6] Bankevich A, Nurk S, Kulikov AS, Prjibelski AD, Tesler G, Vyahhi N, Sirotkin A V., Pham S, Dvorkin M, Nikolenko SI (2012). SPAdes: A new genome assembly algorithm and its application to
single-cell sequencing. J Comput Biol.

[B7] Beneduzi A, Ambrosini A, Passaglia LMP (2012). Plant growth-promoting rhizobacteria (PGPR): Their potential as
antagonists and biocontrol agents. Genet Mol Biol.

[B8] Chaves EID, Guimarães VF, Vendruscolo ECG, dos Santos MF, de Oliveira FF, de Abreu JAC, Camargo MP, Schneider VS, de Souza EM, Cruz LM (2019). Interactions between endophytic bacteria and their effects on
poaceae growth performance in different inoculation and fertilization
conditions. Aust J Crop Sci.

[B9] Chen L, Shi H, Heng J, Wang D, Bian K (2019). Antimicrobial, plant growth-promoting and genomic properties of
the peanut endophyte Bacillus velezensis LDO2. Microbiol Res.

[B10] Chen W, Yang F, Zhang L, Wang J (2016). Organic acid secretion and phosphate solubilizing efficiency of
Pseudomonas sp. PSB12: Effects of phosphorus forms and carbon
sources. Geomicrobiol J.

[B11] Choudhary M, Choudhary BK, Bhoyar S, Kale SB, Chaudhari SP, Bera BC, Jain A, Barbuddhe SB (2018). Isolation and characterization of multidrug-resistant Leclercia
species from animal clinical case. Lett Appl Microbiol.

[B12] Cruz LM, Souza EM, Weber OB, Dobereiner J, Baldani JI, Pedrosa O (2001). 16S Ribosomal DNA characterization of nitrogen-fixing bacteria
isolated from banana (Musa spp.) and pineapple (Ananas comosus (L)
Merril). Appl Environ Microbiol.

[B13] De Werra P, Péchy-Tarr M, Keel C, Maurhofer M (2009). Role of gluconic acid production in the regulation of biocontrol
traits of Pseudomonas fluorescens CHA0. Appl Environ Microbiol.

[B14] Dobereiner J, Baldani VLD, Baldani JI (1995). Como isolar e identificar bactérias diazotróficas de plantas
não-leguminosas.

[B15] Fincheira P, Quiroz A (2018). Microbial volatiles as plant growth inducers. Microbiol Res.

[B16] Finkel OM, Castrilho G, Paredes SH, Gonzales I, Dangl JL (2017). Understanding and exploiting plant beneficial
microbes. Curr Opin Plant Biol.

[B17] Gibeaut DM, Carpita NC (1994). Biosynthesis of plant cell wall polysaccharides. FASEB J.

[B18] Glickmann E, Dessaux Y (1995). A critical examination of the specificity of the Salkowski
reagent for indolic compounds produced by phytopathogenic
bacteria. Appl Environ Microbiol.

[B19] Gupta A, Gopal M, Thomas GV, Manikandan V, Gajewski J, Thomas G, Seshagiri S, Schuster SC, Rajesh P, Gupta R (2014). Whole genome sequencing and analysis of plant growth promoting
bacteria isolated from the rhizosphere of plantation crops coconut, cocoa
and arecanut. PLoS One.

[B20] Horvath P, Barrangou R (2010). CRISPR/Cas, the immune system of Bacteria and
Archaea. Science.

[B21] Hoyos-Mallecot Y, Dolores Rojo-Martín María, Bonnin RA, Creton E, Marí JMN, Naasa T (2017). Draft genome sequence of NDM-1- producing Leclercia
adecarboxylata. Genome Announc.

[B22] Ishino Y, Krupovic M, Forterre P (2018). History of CRISPR-Cas from encounter with a
mysterious. J Bacteriol.

[B23] Jha CK, Saraf G (2015). Plant growth-promoting Rhizobacteria: A critical
review. J Agric Res Dev.

[B24] Kang SM, Shazad R, Bilal S, Khan AL, Park YG, Lee KE, Asaf S, Khan MA, Lee IJ (2019). Indole-3-acetic-acid and ACC deaminase producing Leclercia
adecarboxylata MO1 improves Solanum lycopersicum L. growth and salinity
stress tolerance by endogenous secondary metabolites
regulation. BMC Microbiol.

[B25] Kashani A, Chitsazan M, Che K, Garrison RC (2014). Leclercia adecarboxylata bacteremia in a patient with ulcerative
colitis. Case Rep Gastrointest Med.

[B26] Kochar M, Vaishnavi A, Upadhyay A, Srivastava S (2013). Bacterial biosynthesis of indole-3-acetic acid: signal messenger
service. Mol Microb Ecol Rhizosph.

[B27] Kovacks N (1956). Identification of Pseudomonas pyocyanea by the oxidase
reaction. Nature.

[B28] Laili NS, Othman R, Razarah SS (2017). Isolation and characterization of plant growth-promoting
rhizobacteria (PGPR) and their effects on growth of strawberry (Fragaria
Ananassa Duch.). Bangladesh J Bot.

[B29] Leclerc H (1962). Biochemical study of pigmented Enterobacteriaceae. Ann Inst Pasteur.

[B30] Li M, Guo R, Yu F, Chen X, Zhao H, Li H, Wu J (2018). Indole-3-acetic acid biosynthesis pathways in the
plant-beneficial bacterium Arthrobacter pascens zz21. Int J Mol Sci.

[B31] Loenen WAM, Dryden DTF, Raleigh EA, Wilson GG (2014). Type I restriction enzymes and their relatives. Nucleic Acids Res.

[B32] Moreira FS, Costa PB, De Souza R, Beneduzi A, Lisboa BB, Vargas LK, Passaglia LMP (2016). Functional abilities of cultivable plant growth promoting
bacteria associated with wheat (Triticum aestivum L.) crops. Genet Mol Biol.

[B33] Muratoglu H, Kati H, Demirbag Z, Sezen K (2009). High insecticidal activity of Leclercia adecarboxylata isolated
from Leptinotarsa decemlineata (Col.: Chrysomelidae). J Biotechnol.

[B34] Nautiyal CS (1999). An efficient microbiological growth medium for screening
phosphate solubilizing microorganisms. FEMS Microbiol Lett.

[B35] Nautiyal CS, Bhadauria S, Kumar P, Lal H, Mondal R (2000). Stress induced phosphate solubilization in bacteria isolated from
alkaline soils. FEMS Microbiol Lett.

[B36] Nies DH (2003). Efflux-mediated heavy metal resistance in
prokaryotes. FEMS Microbiol Rev.

[B37] Papenfort K, Bassler B (2014). Quorum-sensing signal-response systems in Gram-negative
bacteria. Nat Rev Microbiol.

[B38] Patten CL, Blakney AJC, Coulson TJD (2013). Activity, distribution and function of indole-3-acetic acid
biosynthetic pathways in bacteria. Crit Rev Microbiol.

[B39] Pierson LS, Pierson EA (2010). Metabolism and function of phenazines in bacteria: impacts on the
behavior of bacteria in the environment and biotechnological
processes. Appl Microbiol Biotechnol.

[B40] Portillo MC, Gonzalez JM (2009). CRISPR elements in the Thermococcales: Evidence for associated
horizontal gene transfer in Pyrococcus furiosus. J Appl Genet.

[B41] Reading NC, Sperandio V (2006). Quorum sensing: The many languages of bacteria. FEMS Microbiol Lett.

[B42] Russell FM, Biribo SSN, Selvaraj G, Oppedisano F, Warren S, Seduadua A, Mulholland EK, Carapetis JR (2006). As a bacterial culture medium, citrated sheep blood agar is a
practical alternative to citrated human blood agar in laboratories of
developing countries. J Clin Microbiol.

[B43] Saitou N, Nei M (1987). The neighbor-joining method: a new method for reconstructing
phylogenetic trees. Mol Biol Evol.

[B44] Sarwar M, Kremer RJ (1995). Determination of bacterially derived auxins using a microplate
method. Lett Appl Microbiol.

[B45] Scagliola M, Pii Y, Mimmo T, Cesco S, Ricciuti P, Crecchio C (2016). Characterization of plant growth promoting traits of bacterial
isolates from the rhizosphere of barley (Hordeum vulgare L.) and tomato
(Solanum lycopersicon L.) grown under Fe sufficiency and
deficiency. Plant Physiol Biochem.

[B46] Schiefner A, Breed J, Bösser L, Kneip S, Gade J, Holtmann G, Diederichs K, Welte W, Bremer E (2004). Cation-pi interactions as determinants for binding of the
compatible solutes glycine betaine and proline betaine by the periplasmic
ligand-binding protein ProX from Escherichia coli. J Biol Chem.

[B47] Sitaraman R (2016). The role of DNA restriction-modification systems in the biology
of Bacillus anthracis. Front Microbiol.

[B48] Souza R de, Ambrosini A, Passaglia LMP (2015). Plant growth-promoting bacteria as inoculants in agricultural
soils. Genet Mol Biol.

[B49] Souza EM, Funayama S, Rigo LU, Pedrosa FDO (1991). Cloning and characterization of the nif A gene from
Herbaspirillurn seropedicae strain Z78. Can J Microbiol.

[B50] Spaepen S, Vanderleyden J (2011). Auxin and plant-microbe interactions. Cold Spring Harb Perspect Biol.

[B51] Suarez C, Ratering S, Hain T, Fritzenwanker M, Goesmann A, Blom J, Chakraborty T, Bunk B, Spröer C, Overmann J (2019). Complete genome sequence of the plant growth-promoting bacterium
Hartmannibacter diazotrophicus Strain E19T. Int J Genomics.

[B52] Suleman M, Yasmin S, Rasul M, Yahya M, Atta BM, Mirza MS (2018). Phosphate solubilizing bacteria with glucose dehydrogenase gene
for phosphorus uptake and beneficial effects on wheat. PLoS One.

[B53] Tamura K, Sakazaki R, Kosako Y, Yoshizaki E (1986). Leclercia adecarboxylata Gen. Nov., Comb. Nov., formerly known as
Escherichia adecarboxylata. Curr Microbiol.

[B54] Thoma R, Hennig M, Sterner R, Kirschner K (2000). Structure and function of mutationally generated monomers of
dimeric phosphoribosylanthranilate isomerase from Thermotoga
maritima. Structure.

[B55] Ueno M, Shibata H, Kihara J, Honda Y, Arase S (2003). Increased tryptophan decarboxylase and monoamine oxidase
activities induce Sekiguchi lesion formation in rice infected with
Magnaporthe grisea. Plant J.

[B56] Yan L, Allen MS, Simpson ML, Sayler GS, Cox CD (2007). N-(3-oxo-hexanoyl)-L-homoserine lactone (3-oxo-C6-HSL) plays a
significant role as autoinducer in the quorum-sensing system. J Microbiol Methods.

[B57] Yano DMY, Attili DS, V GMS, Eguchi SY, Oliveira UM (1991). Técnicas de microbiologia em controle de qualidade.

[B58] Yi HS, Ahn YR, Song GC, Ghim SY, Lee S, Lee G, Ryu CM (2016). Impact of a bacterial volatile 2,3-butanediol on Bacillus
subtilis rhizosphere robustness. Front Microbiol.

