# Supplementary material for: Genome sequencing and analysis of plant growth-promoting attributes from Leclercia adecarboxylata
Source: Genet Mol Biol. 2021 Jan 27;44(1):e20200130. doi: 10.1590/1678-4685-GMB-2020-0130 (PMC7839631; doi:10.1590/1678-4685-GMB-2020-0130)
Supplement: Figure S1 - [file 1415-4757-GMB-44-1-e20200130-s3.pdf]

Genetics and Molecular Biology
https://doi.org/10.1590/1678-4685-GMB-2020-0130

Supplementary material to "Genome sequencing and analysis of plant growth-promoting attributes from *Leclercia adecarboxylata*"

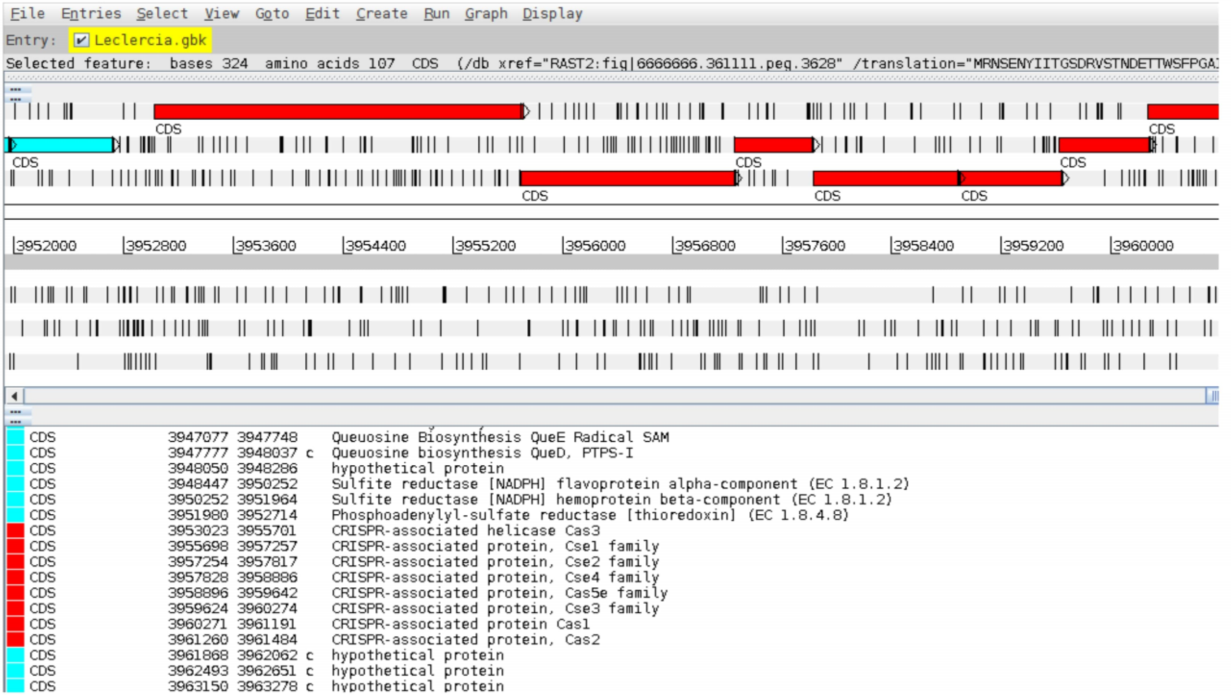

Figure S1 - Genomic map of CRISPR system
